# Remission of low-grade lymphomatoid granulomatosis with extensive pulmonary involvement following immune restoration via antiretroviral therapy in a newly diagnosed HIV patient

**DOI:** 10.1186/s12981-025-00717-9

**Published:** 2025-02-15

**Authors:** Maria Kogan, Antonio Maria Alviano, Martina Catalano, Alessandra Casiraghi, Giulia Ghilardi, Giovanni Rindone, Luisa Verga, Vincenzo L’Imperio, Carlo Gambacorti Passerini, Paolo Bonfanti, Giuseppe Lapadula, Federica Cocito, Alessandro Soria

**Affiliations:** 1 School of Medicine, University of Milano-Bicocca, Monza, Italy; 2 Clinic of Haematology, Fondazione IRCCS San Gerardo dei Tintori, Monza, Italy; 3 Pathology Department, Fondazione IRCCS San Gerardo dei Tintori, Monza, Italy; 4 Clinic of Pneumology, Fondazione IRCCS San Gerardo dei Tintori, Monza, Italy; 5 Unit of Radiodiagnostic, Fondazione IRCCS San Gerardo dei Tintori, Monza, Italy; 6 Clinic of Infectious Diseases, Fondazione IRCCS San Gerardo dei Tintori, Monza, Italy

**Keywords:** Lymphomatoid granulomatosis, Immune recovery, HIV, Lung infiltrates, Low-grade histology

## Abstract

Lymphomatoid granulomatosis (LYG) is a rare Epstein-Barr virus (EBV)-driven lymphoproliferative disease that usually arises in the context of reduced immunological surveillance. Based on histology, two forms of the disease are recognized, namely low-grade and high-grade LYG. Clinically, LYG universally involves the lungs and, frequently, also the skin, central nervous system, liver, and kidneys. Here, we present the case of a 55-year-old woman with a difficult-to-diagnose low-grade LYG with symptomatic lung involvement, who concomitantly was newly diagnosed with human immunodeficiency virus (HIV) infection. Rapid immune recovery achieved through antiretroviral therapy led to a complete and sustained clinical and radiological remission of LYG.

## Introduction

Lymphomatoid granulomatosis (LYG) is a rare Epstein-Barr virus (EBV)-driven lymphoproliferative disease [1] typically affecting patients with an underlying immunodeficiency that impairs EBV immune surveillance and viral clearance. The disease is generally extranodal, with infiltrative lesions most commonly affecting the lungs. Other common extranodal sites include the skin, central nervous system, and kidneys, while the bone marrow and lymph nodes are rarely involved. This uncommon lymphoproliferative disorder poses a significant diagnostic challenge due to its rarity, heterogeneous behavior, and scarce available clinical data.

LYG has unique histopathologic and clinical features which are distinct from other EBV-related B-cell lymphoproliferative diseases and lymphomas. In 2001, LYG was incorporated into the World Health Organization classification of Tumors of Hematopoietic and Lymphoid Tissues [2], and remains in the 2022 5^th^ Edition as a distinct mature B-cell neoplasm [3].

Based on histology, three grades of LYG are recognized, depending on the number and density of EBV-positive B-cells and the degree of coagulative necrosis: grades 1 and 2 correspond to low-grade disease, while grade 3 is high-grade disease [4]. The current therapeutic strategy is based on LYG histological classification: low-grade disease is typically polyclonal and thus is assumed to be immune dependent. Therefore, treatment focuses on enhancing the host immune response, for example with the use of alpha-interferon and intravenous gamma-globulin. Chemotherapy and systemic corticosteroids, although effective in reducing organ lesions, failed to provide long term disease control in low-grade LYG [1]. Thus, after withdrawal of potential iatrogenic causes of immunosuppression, low-grade LYG may be initially treated with a “watch and wait” approach; however, most cases eventually require treatment, as the progression rate to high-grade overt lymphoma remains high [5].

Conversely, treatment of high-grade LYG typically requires immunochemotherapy, using aggressive non-Hodgkin lymphoma protocols [6]. High-grade LYG is considered an aggressive disease, with an estimated median overall survival of less than two years [7].

## Case presentation

**Fig. 1:**
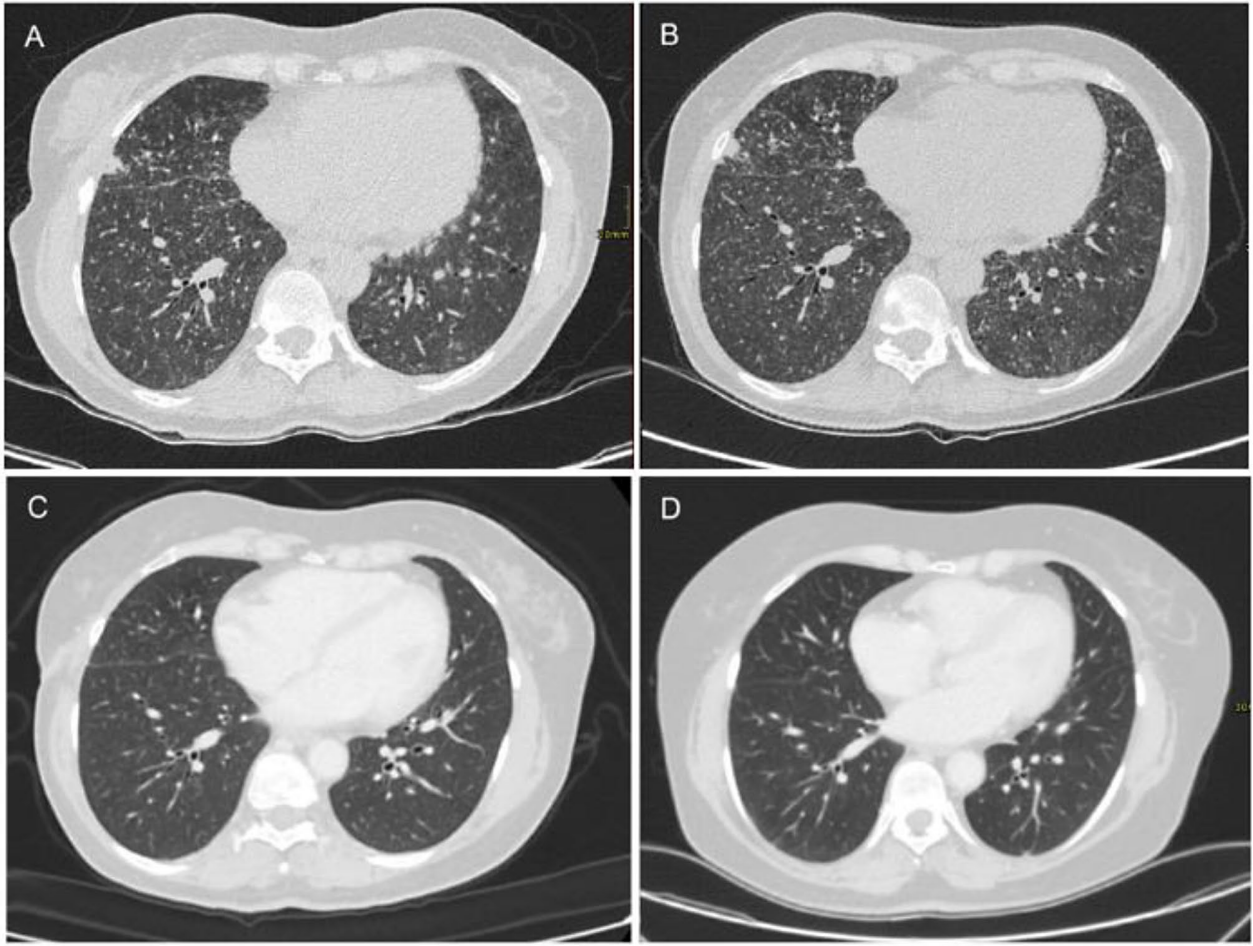
Serial computed tomography scans showing progressive resolution of the diffuse micronodular interstitial pattern of the lungs following initiation of antiretroviral therapy on 16 November 2022 (**A**: 26 November 2021; **B**: 22 September 2022; **C**: 30 January 2023; **D**: 30 May 2024)

A previously healthy 55-year-old woman presented to her primary care physician in August 2021 with a history of persistent low-grade fever, peripheral edema and night sweats. Blood tests were normal except for high immunoglobulin levels. Chest radiograph showed diffuse bilateral lung micronodules, so the patient was referred to a pulmonology specialist for further investigations in December 2021. A chest computed tomography (CT) scan confirmed a bilateral diffuse micronodular interstitial pattern, predominantly in the lower lung lobes. In addition, small subpleural nodules with areas of ground glass opacities and enlarged mediastinal lymph nodes were documented (max diameter 2 cm, Fig. 1A).

Spirometry showed a restrictive pattern with reduction of diffusing capacity for carbon monoxide. Echocardiogram showed a normal systolic function, the absence of valve abnormalities and a normal right heart. Fibrobronchoschopy did not reveal abnormal findings. Broncholveolar lavage for microbiological investigations (including research for tuberculosis) turned up negative and cytological exam resulted negative for neoplastic cells; cell count showed 72% alveolar macrophages, 18% neutrophils, 10% lymphocytes, with the following immunophenotyping: B lymphocytes 9% (112/mm^3^), CD4+ T-lymphocytes 4% (45/mm^3^), CD8+ T-lymphocytes 88% (1113/mm^3^), CD3+ T lymphocytes 91% (1143/mm^3^), natural killer lymphocytes 0.4% (5/mm^3^), T4/T8 ratio 0.04, total lymphocyte count 1261/mm^3^. Transbronchial biopsy demonstrated a nonspecific histology consisting of B-lymphocyte aggregates associated with isolated CD30+ elements and a positive EBV-encoded small RNAs (EBER) in-situ hybridization (ISH). Ziehl-Neelsen stain was negative.

A new CT scan was subsequently performed in February 2022 (Fig. 1B). It confirmed the previously described pulmonary findings and showed supraclavicular, mediastinal, inguinal, and retroperitoneal lymphadenopathy (max diameter 27 × 13 mm). Histopathological analysis of an inguinal lymph node biopsy revealed a conserved nodal architecture with CD3+/CD5+ T cells in the paracortical region and hyperplastic lymphoid follicles containing CD20+/CD10+/BCL6+/BCL2- B lymphocytes in the cortical area, consistent with reactive follicular hyperplasia.

A repeated transbronchial biopsy showed lung tissue with septal infiltrate of B (CD20+) and T (CD3+) lymphocytes, and plasma cells. No CD30+ nor IgG4+ elements were observed. PAS, Grocott and Ziehl-Neelsen stains were negative.

**Fig. 2:**
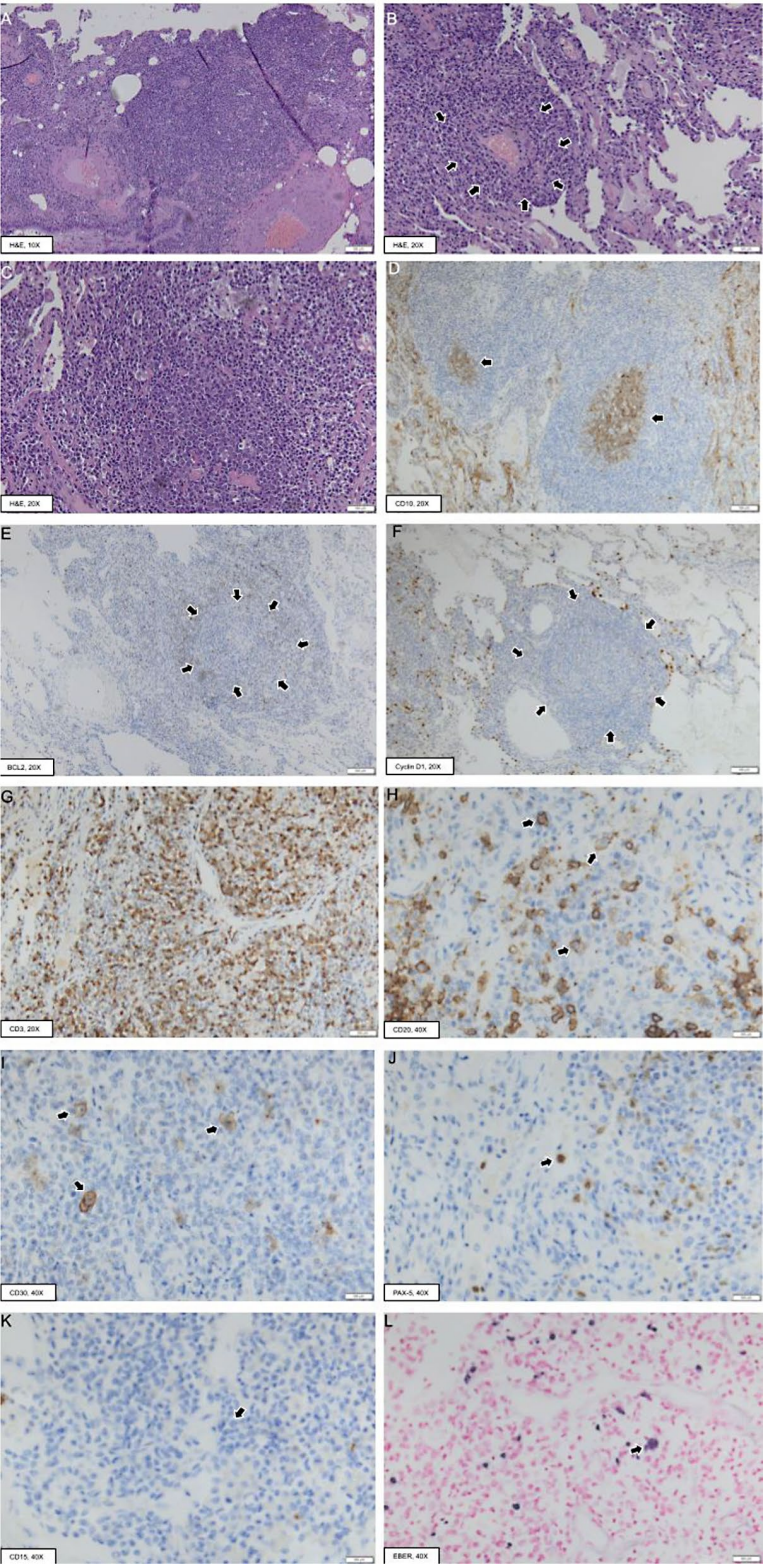
Representative histopathological images of the surgical lung biopsy material. Light microscopy examination showed perivascular aggregates of lymphoplasmacytic elements (**A-C**), with focal angioinvasion (delimited by the arrows in **B**). Scattered lymphoid follicles were noted, with CD10+/BCL2- germinal centers (arrows in **D** and **E**, respectively) and Cyclin D1- mantle zones (delimited by the arrows in **F**). An abundant T-cell population (CD3+) was also present (**G**). This florid cellular infiltrate contained medium to large-sized atypical lymphoid elements, which were found to be CD20+ (**H**, arrows) / CD30+ (**I**, arrows) / PAX-5+ (**J**, arrow) / CD15- (**K**, arrow) / EBER+ (**L**, arrow). EBER, Epstein-Barr virus - Encoded small RNAs. H&E, Hematoxylin&Eosin

Upon worsening of dyspnea in October 2022, the patient consented to a surgical lung biopsy. Histological examination showed a lymphoplasmacytic infiltrate organized in perivascular aggregates with focal angioinvasion (Fig. 2A-C). Occasional lymphoid follicles were noted which contained a CD23+ dendritic cell meshwork, polarized germinal centers (CD10+/BCL2-) (Fig. 2D-E), and Cyclin D1- mantle zones (Fig. 2F). Immunohistochemical staining for the CD3 marker showed an abundant population of T lymphocytes throughout the interstitial infiltrate and in the regions surrounding the lymphoid follicles (Fig. 2G). Scattered CD79a+/Human Herpesvirus 8 (HHV8)- plasma cells were also present, with no evidence of immunoglobulin light chain restriction. Notably, the interstitial infiltrate contained up to 4–5 atypical lymphoid elements of intermediate/large size per high-power field (HPF), which were found to be CD20+, CD30+, PAX5+, CD15-, ALK-, and EBER+ (Fig. 2H-L). Thus, histology was compatible with an EBV-related B-cell lymphoproliferative disease with characteristics of low-grade (G1-G2) LYG.

In November 2022, the patient was referred to the hematology unit for management. Laboratory tests in the staging work-up included human immunodeficiency virus (HIV) serology, (eventually accepted by the patient after repeated refusal in the previous year), which yielded a positive result. The patient’s initial CD4+ T lymphocyte count was 150 cells/mm^3^, and plasma HIV RNA level was 363,000 copies/ml. She was referred to the infectious diseases unit and was started on antiretroviral therapy with coformulated emtricitabine/tenofovir alafenamide/bictegravir, achieving undetectable viral load within four months and obtaining rapid immune recovery to 494 CD4+ cells/mm^3^.

A positron emission tomography scan performed before starting antiretroviral therapy demonstrated active disease in the rhinopharynx, lungs, and lymph nodes (both above and below the diaphragm), with a max standardized uptake value of 13 MBq/gr.

To reduce the risk of infections in the setting of newly diagnosed advanced HIV disease, immunochemotherapy for LYG was initially postponed for a few months. At the first follow-up visit in January 2023, the patient showed significantly improved conditions, with 3-kg weight gain, and reduced dyspnea. Blood EBV DNA level dropped from 23,627 to 3,116 copies/ml.

In January 2023, three months after starting antiretroviral therapy, the CT scan showed a clear regression of the lung lesions (Fig. 1C). Considering the rapid clinical and radiological improvement, we decided to withhold immunochemotherapy. In October 2023, a new CT scan showed complete regression of the pulmonary lesions, while pulmonary function tests normalized. These results were confirmed in May 2024 (Fig. 1D), thus achieving a complete and sustained clinical and radiological remission with the sole antiretroviral therapy. The last available clinical and laboratory follow-up (December 2024, two years after HIV diagnosis) confirmed the absence of indication to start immunochemotherapy for LYG.

## Discussion

LYG is generally considered an aggressive EBV-related condition, as the majority of patients develop progressive disease in the absence of treatment. However, the clinical course of the disease can be quite variable: this is reflected in the distinction between high- and low-grade forms, based on the number and density of EBV+ B cells and the degree of coagulative necrosis. Low-grade LYG is typically diagnosed in patients with reduced immunological surveillance: if the latter is restored, a good control of the disease can be achieved. In our patient, baseline immunodeficiency was determined by HIV infection, which was unknown at the time of initial presentation and was diagnosed only after the standard work-up for lymphoproliferative disorders. Despite the extent of the disease at diagnosis, with multiple lung infiltrates threatening pulmonary function, we observed a complete and sustained remission of both clinical and radiological findings along with the progressive immune recovery obtained with antiretroviral therapy, with no need of further treatment. This case suggests that in the therapeutic management of low-grade LYG it could be preferable to first privilege immunological control, rather than immediately starting immunochemotherapy. In addition, after a new LYG diagnosis, the underlying cause of immunodeficiency should be sought, especially if potentially reversible, as is the case for advanced HIV infection with antiretroviral therapy.

Variable outcomes have been reported in the literature for patients with HIV-associated LYG, depending on the main localization of the lymphoproliferative process [8–11]. For instance, complete remission with antiretroviral therapy alone has been observed in a patient with predominant skin localization [10], while a severe presentation has been reported in the case of central nervous system involvement [9]. The effect of immune restoration due to antiretroviral therapy on the control of other viruses establishing latency, including EBV, is thought to be crucial in determining the remission of low-grade LYG with pulmonary involvement, as shown in another case [8].

Our case also highlights that late HIV diagnosis still occurs in Western World. One common cause is not perceiving oneself at risk, thus considering outrageous the request to be tested for HIV. This was a limitation of our diagnostic process, as it determined a delay of one and half year from the first nonspecific symptoms to HIV diagnosis. Although lymphoproliferative disorders are a frequent presentation of a new HIV diagnosis, HIV-associated LYG is quite rare. Thus, clinicians should be aware of this unusual presentation of low-grade LYG associated with advanced HIV infection and maintain a high index of suspicion, in order to promptly implement a successful therapeutic strategy.

## Data Availability

No datasets were generated or analysed during the current study.
